# A novel likely pathogenic *CLCN5* variant in Dent’s disease

**DOI:** 10.1186/s12882-023-03292-1

**Published:** 2023-08-28

**Authors:** S Hayward, J Norton, L Bownass, C Platt, Genomics England Research Consortium, J. C. Ambrose, P Arumugam, R. Bevers, M. Bleda, F. Boardman-Pretty, C. R. Boustred, H. Brittain, M. A. Brown, M. J. Caulfield, G. C. Chan, A. Giess, J. N. Griffin, A. Hamblin, S. Henderson, T. J. P. Hubbard, R. Jackson, L. J. Jones, D. Kasperaviciute, M. Kayikci, A. Kousathanas, L. Lahnstein, A Lakey, S. E. A. Leigh, I. U. S. Leong, F. J. Lopez, F. Maleady-Crowe, M. McEntagart, F. Minneci, J. Mitchell, L. Moutsianas, M. Mueller, N. Murugaesu, A. C. Need, P. O‘Donovan, C. A. Odhams, C. Patch, D. Perez-Gil, M. B. Pereira, J. Pullinger, T. Rahim, A. Rendon, T. Rogers, K. Savage, K. Sawant, R. H. Scott, A. Siddiq, A. Sieghart, S. C. Smith, A. Sosinsky, A. Stuckey, M. Tanguy, A. L. Taylor Tavares, E. R. A. Thomas, S. R. Thompson, A. Tucci, M. J. Welland, E. Williams, K. Witkowska, S. M. Wood, M. Zarowiecki, H Campbell, E Watson, N Forrester, S Smithson, A Menon

**Affiliations:** 1 Bristol Medical School, Translational Health Sciences, University of Bristol, Bristol, UK; 2 Richard Bright Renal Service, Southmead Hospital, North Bristol NHS Trust, Bristol, UK; 3 South West Genetic Laboratory Hub, North Bristol NHS Trust, Bristol, UK; 4 Department of Clinical Genetics, University Hospitals Bristol and Weston NHS Foundation Trust, Bristol, UK; 5 Department of Paediatric Nephrology, University Hospitals Bristol and Weston NHS Foundation Trust, Bristol, UK; 6 Genomics England Research Consortium, Genomics England, London, UK

**Keywords:** Dent’s disease, Genetics, Nephrocalcinosis, Proteinuria, *CLCN5*

## Abstract

**Background:**

The majority of cases of Dent’s disease are caused by pathogenic variants in the *CLCN5* gene, which encodes a voltage-gated chloride ion channel (ClC-5), resulting in proximal tubular dysfunction. We present three members of the same family and one unrelated paediatric patient with the same insertion-deletion *CLCN5* variant. The identification of these patients and positive familial segregation led to the re-classification of this variant from one of unknown significance to one of likely pathogenicity.

**Case presentation:**

A 41 year old male presented with end stage kidney failure, proteinuria and haematuria. Whole genome sequencing identified an insertion-deletion variant in *CLCN5*, resulting in a missense change (c.1744_1745delinsAA p.(Ala582Lys)). His brother and nephew, who both exhibited renal impairment, haematuria, proteinuria, glycosuria and nephrocalcinosis, were found to have the same variant. In addition, genetic testing of an unrelated paediatric patient who presented with proteinuria and hypercalciuria, demonstrated the same variant.

**Conclusions:**

The identification of this novel variant in four individuals with features of Dent’s disease, has led to the re-classification of the variant to one of likely pathogenicity.

As a result, our patients and any future patients with the same variant can be offered a likely diagnosis, without the need for kidney biopsy, and their family members can be offered genetic screening.

## Background

Dent‘s disease is an X-linked recessive condition leading to dysfunction of the renal proximal tubule. The most common clinical phenotype is nephrocalcinosis (secondary to hypercalciuria), proteinuria (predominantly low molecular weight proteins) and renal impairment [1]. However, patients can also exhibit a Fanconi type picture of tubular wasting, with glycosuria and aminoaciduria. In addition, some patients present with skeletal problems linked to rickets or osteomalacia, due in part to urinary loss of vitamin D. During adulthood, patients may develop progressive chronic kidney disease leading eventually to the need for renal replacement therapy [1].

The majority of people with Dent’s disease have pathogenic variants in the *CLCN5* (Dent disease 1, roughly 60% of patients) or *OCRL1* genes (Dent disease 2, roughly 16% of patients) [2]. The genetic basis in the remaining patients is yet to be identified (Dent disease 3) [3]. The *CLCN5* gene encodes the chloride channel Cl-/H+ exchanger ClC-5 in the kidney, which is required for low-molecular-weight protein uptake in the renal proximal tubule [4]. At least 266 pathogenic variants in *CLCN5* have been previously described [4].

We report the identification of an insertion-deletion *CLCN5* variant in three members of the same family, as well as one unrelated paediatric patient with Dent’s disease. Our findings have led to the re-classification of this *CLCN5* variant from one of unknown significance to one of likely pathogenicity [5], enabling early diagnosis and pre-symptomatic testing of family members if requested.

## Case presentation

### First index case

A 41-year-old male presented with back pain, nausea and vomiting. His only past medical history was of gastro-oesophageal reflux disease. He had been taking ibuprofen and tramadol for two weeks prior to his presentation but was on no other regular medications. Examination demonstrated a slightly raised blood pressure (Table 1) but was otherwise unremarkable. His blood and urine tests showed evidence of renal failure, proteinuria, and haematuria (summarised in Table 1). A renal ultrasound revealed small kidneys (right 8.9 cm and left 8.8 cm) with reduced corticomedullary differentiation and several small simple cysts. He underwent a renal biopsy which showed significant interstitial fibrosis and tubular atrophy, with calcium and neutrophils present in the tubules on light microscopy. A twenty-four hour urine collection was performed but only yielded a urine volume of 0.5 litres; the results are listed in Table 1 but should be interpreted with caution as the urine collection volume was insufficient. His diagnosis was uncertain, he was commenced on haemodialysis and subsequently went on to receive a transplant.

**Table 1:** Investigation results at the time of presentation

**Test**	**1**^**st**^** index case**	**Brother**	**Nephew**	**2**^**nd**^** index case**	**Adult reference range**
Serum creatinine (umol/L)	1026	257	163	39	59 - 104
Blood urea nitrogen (mmol/L)	26.4	12.6	6.1	3.9	2.5 – 7.8
Serum phosphate (mmol/L)	1.17	0.85	0.69	1.5	0.8 – 1.5
Serum adjusted calcium (mmol/L)	1.73	2.45	2.42	2.53	2.2 – 2.6
Serum parathyroid hormone (pmol/L)	68.7	3.7	2.2	0.8	1.6 – 6.9
Urinalysis	Blood 2+ Protein 1+	Blood 1+ Protein 1+ Glucose 1+	Blood 4+ Protein 1+ Glucose 2+	Protein 3+	N/A
Urine protein: creatinine ratio (mg/mmol)	256	164	94	250	< 50
Urine phosphate (mmol/sample)	2.4	Not performed	15.6	Not performed	15.0 – 50.0
Urine calcium (mmol/sample)	0.2	Not performed	5.2	0.93^a^	2.5 – 7.5^a^
Urine sodium (mmol/sample)	17	Not performed	184	80	50 – 200
Urine oxalate (mmol/sample)	86	Not performed	277	Not performed	100 – 460
Blood pressure (mm/Hg)	140/90	138/80	102/60	91/57	N/A

^a^Paediatric reference range for urinary calcium < 0.7 mmol/sample.

### Other family members

**Fig. 1:**
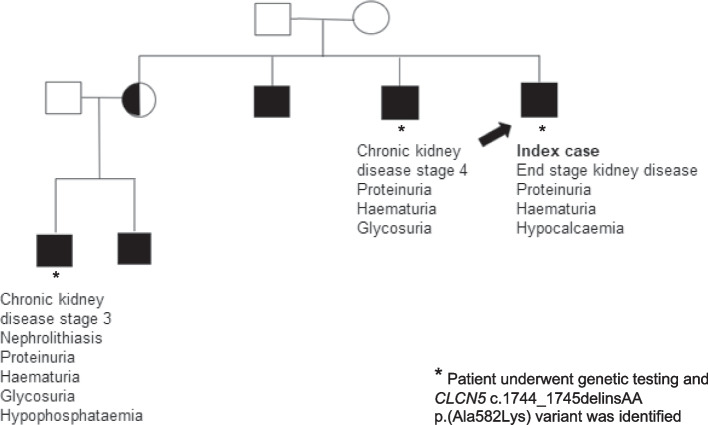
Family tree 1

Over time further male family members were identified as having a similar phenotype as the first index case, including a brother and a nephew (Figure 1). The brother was referred to Nephrology at age 37 after routine screening discovered renal impairment (Table 1). He had a past medical history of gastro-oesophageal reflux disease and was taking Rabeprazole 10mg once a day. His investigations showed stage 4 chronic kidney disease with haematuria, proteinuria and glycosuria; his blood pressure, blood and urine test results are summarised in Table 1. A renal ultrasound was unremarkable demonstrating 9.4 cm right kidney and 9.3 cm left kidney. He underwent a renal biopsy which showed calcium deposition in the medulla on light microscopy and immunofluorescence was positive for IgA deposition. He was not given a diagnosis for his underlying renal condition.

The nephew, aged 32, was referred to the Metabolic stones renal clinic with nephrolithiasis. His only past medical history was renal calculi and he took no regular medications. His examination was unremarkable. He had evidence of stage 3 chronic kidney disease with mild hypophosphataemia, haematuria, proteinuria, and glycosuria. His blood pressure, blood and urine test results are summarised in Table 1. A Computerised Tomography scan of his kidneys demonstrated multiple bilateral renal stones and no hydronephrosis. A renal biopsy showed calcium and phosphate crystals in the tubules and interstitium on light microscopy and immunofluorescence was negative for IgA. His mother (sister of the index case) had been seen by renal services previously with a history of chronic pyelonephritis. On imaging, she was found to have grade 1 reflux disease.

### Genetic testing

Once the family history was identified, the first index case underwent whole genome sequencing (WGS) as part of the 100,000 Genomes Project. DNA was extracted from a peripheral blood sample following standard WGS protocols, approved by the 100,000 Genomes Project. Relevant guidelines and regulations were adhered to. WGS identified a hemizygous insertion-deletion variant resulting in a missense change in the *CLCN5* gene (c.1744_1745delinsAA p.(Ala582Lys), situated on the X chromosome at Xp11.23, which was initially classified as being of unknown significance. Both his brother and nephew underwent genetic testing as part of their routine clinical care and were positive for the same *CLCN5* variant. The variants were confirmed with Sanger sequencing. Other male family members were identified, corresponding to an X-linked inheritance pattern, but chose not to be tested. The sister of the index case (nephew’s mother) did not undergo genetic testing. Female carriers of pathogenic variants of the *CLCN*5 gene can in some cases exhibit a milder phenotype such as low level proteinuria, hypercalciuria and rarely kidney stones [6]. No urine testing for calcium or proteinuria was carried out in this case.

### Second index case

**Fig. 2:**
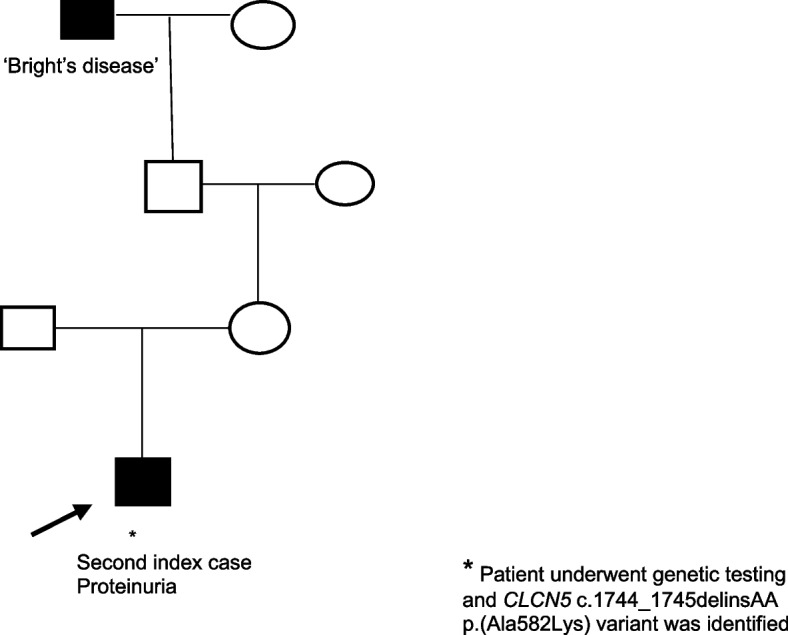
Family tree 2

In the interim, an unrelated male paediatric patient in our region had undergone genetic testing and was found to have the same *CLCN5* variant. He presented aged 7 years and 5 months with an incidental finding of proteinuria after an episode of sinusitis with a facial nerve palsy. He had no additional past medical history. His maternal great great grandfather had been diagnosed with ‘Bright’s disease’ but there was no other family history of note (Figure 2). His blood pressure, blood and urine test results are presented in Table 1. He underwent a renal biopsy which showed ‘tip variant’ focal segmental glomerulosclerosis, understood to be associated with a milder disease course [7].

### Variant re-classification

The pathogenicity of the *CLCN5* sequence variant was interpretated according to the American College of Medical Genetics and Genomics (ACMG) and the Association for Clinical Genomic Science (ACGS) best practice guidelines [5, 8]. The identification of the same variant in the unrelated second index case, alongside the segregation information from the first index case’s family, allowed our team to re-classify the *CLCN5* variant (c.1744_1745delinsAA p.(Ala582Lys) from one of unknown significance to one of likely pathogenicity (Table 2) [5, 9]. The ACMG/ACGS codes used for this re-classification were: PS4-Supporting, PM2-Moderate, PP1-Supporting, PP3-Supporting and PP4-Supporting (Table 2).

**Table 2:** Evidence for classification of the *CLCN5* c.1744_1745delinsAA p.(Ala582Lys) variant using ACMG and ACGS guidelines [5, 8]

**Criteria code weight**	**Pathogenic evidence**
**PS4 Supporting**	*The prevalence of the variant in affected individuals is significantly increased compared with the prevalence in controls*. The variant was identified in unrelated index patients affected with X-linked Dent disease in the Bristol Genetics Laboratory.
**PM2 Moderate**	*Absent from controls (or at extremely low frequency if recessive)*. Not recorded on the population database gnomAD.
**PP1 Supporting**	*Co-segregation with disease in multiple affected family members in a gene definitively known to cause the disease*. Observed segregating in at least three informative meioses in a family.
**PP3 Supporting**	*Multiple lines of computational evidence support a deleterious effect on the gene or gene product*. Polyphen: Probably damaging; SIFT: Deleterious; Splicing prediction tools: No effect; Align AGVD: C65; Amino acid conservation: High.
**PP4 Supporting**	*Patient’s phenotype or family history is highly specific for a disease with a limited number of genetic aetiologies.* The second index patient with this variant is reported to have clinical Dent’s disease, which is distinctly associated with the *CLCN5* gene, where other known genetic aetiologies have been excluded.
**Summary**	**1x Moderate and 4x Supporting = Likely pathogenic**

## Discussion and conclusions

We found a novel insertion-deletion variant in the *CLCN5* gene in three individuals from the same family and one unrelated paediatric case with Dent’s disease, resulting in the variant being re-classified to likely pathogenic. The Dent’s disease phenotype is defined as the presence of low-molecular weight proteinuria, hypercalciuria and at least one of hypophosphataemia, nephrocalcinosis, nephrolithiasis, haematuria, or renal impairment [10]. However, there can be significant phenotypic heterogeneity. Many patients with pathogenic *CLCN5* or *OCRL* variants can exhibit incomplete Dent’s phenotypes [11]. Indeed, even *CLCN5* knock-out mouse models, which result in a total loss of functional protein, can demonstrate diverse phenotypes. For example, *CLCN5* knock out mice can develop hyperphosphaturia, hypercalciuria and nephrocalcinosis, whereas others only exhibit hyperphosphaturia [12, 13]. Our cases further illustrate the breadth of clinical features and age at presentation of Dent’s disease, even in people with the same underlying genetic variant, suggesting that genetic background and other factors interact to influence the phenotype. All our related cases demonstrated renal impairment, haematuria, proteinuria and nephrocalcinosis. However, presence of the other typical features of Dent’s disease varied; only one of the cases had symptomatic renal calculi and hypophosphataemia, and none exhibited hypercalciuria. The index case had renal cysts, which have been reported in 30% of cases of Dent’s disease [6]. In contrast, the unrelated paediatric patient demonstrated only proteinuria and hypercalciuria.

The majority of *CLCN5* disease-causing variants that have been identified are missense, frameshift or nonsense mutations (35%, 31% and 16%, respectively) [4]. In-frame insertion-deletions, like the variant we identified, are relatively rare in Dent’s disease, accounting for only 2% of pathogenic *CLCN5* variants. Through the identification of the insertion-deletion variant in two different generations of the same family and the addition of a second index case with Dent’s disease, we were able to re-classify this variant from one of unknown significance to one of likely pathogenicity. Genetic investigation is becoming more prevalent in clinical practice, including testing patients with incomplete Dent’s phenotypes. In addition, pathogenicity prediction algorithms and other methods to discover the protein consequences of variants are also being improved. Therefore, it is plausible that in the next decade we will improve the interpretation of variants within *CLCN5* or *OCRL *.

For roughly 25% of people with Dent’s disease, no pathological variant in *CLCN5* or *OCRL* is found, and patients are classified as having Dent disease 3 [14], indicating further locus heterogeneity. Studies which have focused on identifying variants in other genes on the X chromosome in people with Dent disease 3 have yielded little success. Candidate genes which have been explored and produced negative results include: *CLCN4,* a member of the same family of chloride channels as *CLCN5* [2]*; SLC9A6*, which encodes a sodium-hydrogen exchanger [2]; and *CLTRN,* which encodes collectrin, a transmembrane protein that mediates amino acid transport in the proximal tubule [15]. One promising study which investigated outwith the X chromosome performed exome sequencing on eight patients with Dent disease 3. The study detected variants of unknown significance in *SLC9A3* (chromosome 5) and *PDZK1* (chromosome 1). These variants were predicted to be either disease causing or damaging by in silico tools and warrant further investigation [16]. At present, conclusive evidence of pathogenic variants in genes other than *CLCN5* or *OCRL* remains elusive in Dent’s disease.

The structure and organisation of our regional genetics service was crucial to identifying the same variant in unrelated patients. The service includes a virtual monthly multi-disciplinary team meeting attended by both adult and paediatric renal genetics teams, allowing for detailed case discussions and sharing of information. Prior to genetic testing, the three related patients had no clear diagnosis. Diagnostic uncertainty places a significant psychological burden on patients and can lead to over-investigation [17]. Also, in Nephrology, this uncertainty raises unanswerable questions about the risk of disease recurrence after transplantation. As a result of the genetic testing, we could offer these patients a likely diagnosis and initiate appropriate monitoring and treatment. In addition, we could offer genetic counselling and testing to other members of their family at risk of the condition. Furthermore, in the future, if any other family members present with a similar phenotype, they could undergo genetic testing and avoid the need for a kidney biopsy, which is an invasive procedure that can result in serious complications.

To conclude, the identification of the novel insertion-deletion *CLCN5* variant in four individuals with renal impairment, proteinuria and haematuria, has led to the re-classification of the variant to one of likely pathogenicity for Dent’s disease. Our findings extend the spectrum of known *CLCN5* variants and emphasise the phenotypic heterogeneity of Dent’s disease. As a result of the re-classification of the variant, our patients and any future patients with the same variant can be offered a likely diagnosis, without the need for kidney biopsy, and genetic screening for other family members.

## Data Availability

The datasets generated and analysed during the current study are not publicly available to protect the privacy of the patients. The corresponding author (AM) can be contacted to discuss data access.

## Abbreviations

WGS: Whole genome sequencing
ACMG: American College of Medical Genetics and Genomics
ACGS: Association for Clinical Genomic Science
